# CK 20‐positive and CK 7‐negative Merkel cell carcinoma of the cheek

**DOI:** 10.1002/ccr3.1383

**Published:** 2018-02-01

**Authors:** Manoj Ponadka Rai, Prabhjot S Bedi, Jeevandeep Singh, Edwin B. Marinas

**Affiliations:** ^1^ Michigan State University/Sparrow Hospital 788 Service Road B301 Clinical Center East Lansing Michigan 48824; ^2^ UPMC East 2775 Mosside Blvd Monroeville Pennsylvania 15146; ^3^ Sparrow Hospital 1215 E Michigan Ave Lansing Michigan 48912

**Keywords:** CK 20‐positive, CK 7‐negative, Merkel cell carcinoma, polyomavirus

## Abstract

Merkel cell carcinomas (MCCs) are uncommon, highly malignant skin tumors that develop in sun‐exposed areas of the skin. Most of the MCCs are CK 20‐positive and CK 7‐negative such as our case. About 80% of Merkel cell carcinoma is associated with Merkel cell polyomavirus.

## Case

**Figure 1:**
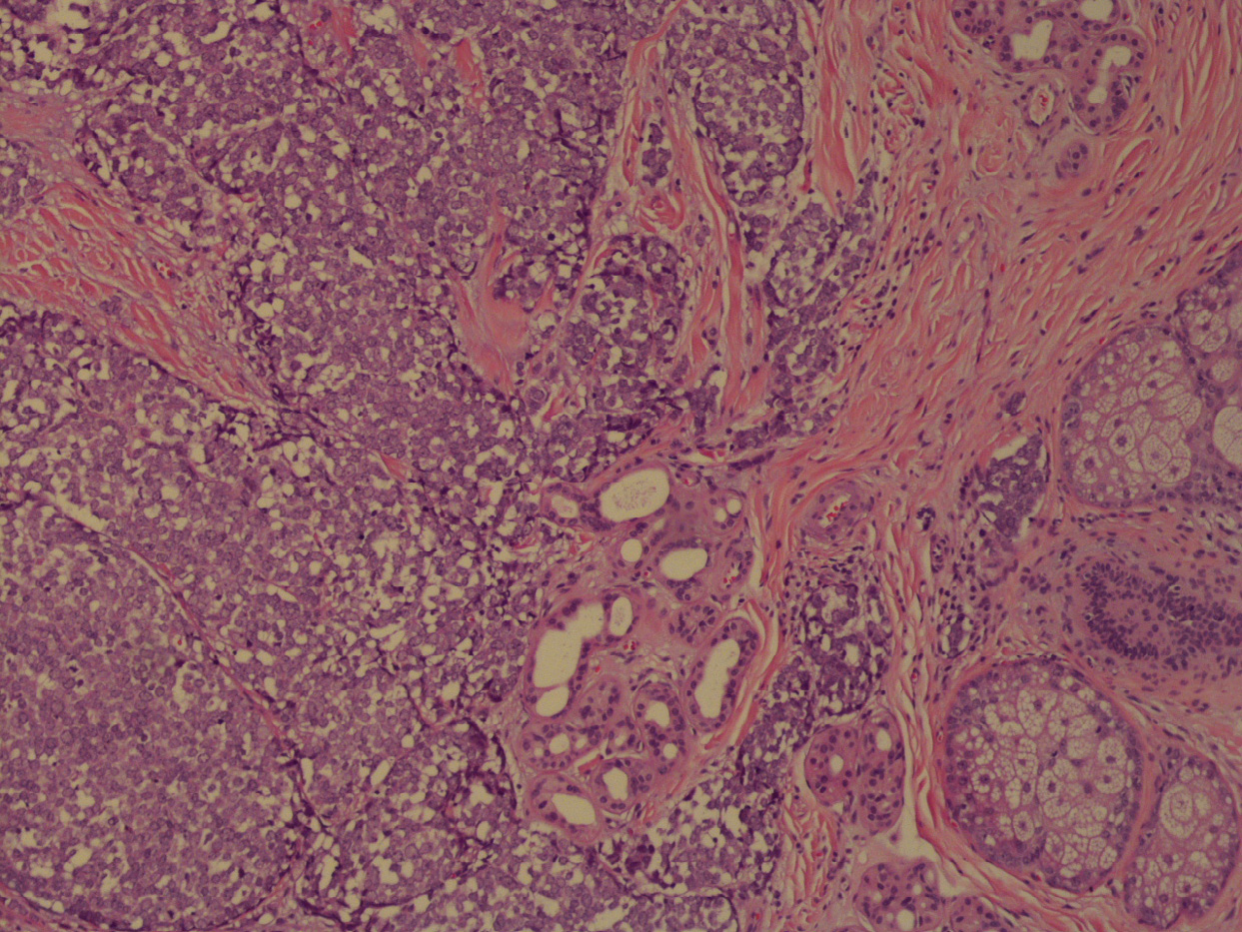
Histopathology (low power resolution) showing neuroendocrine features of the tumor.

**Figure 2:**
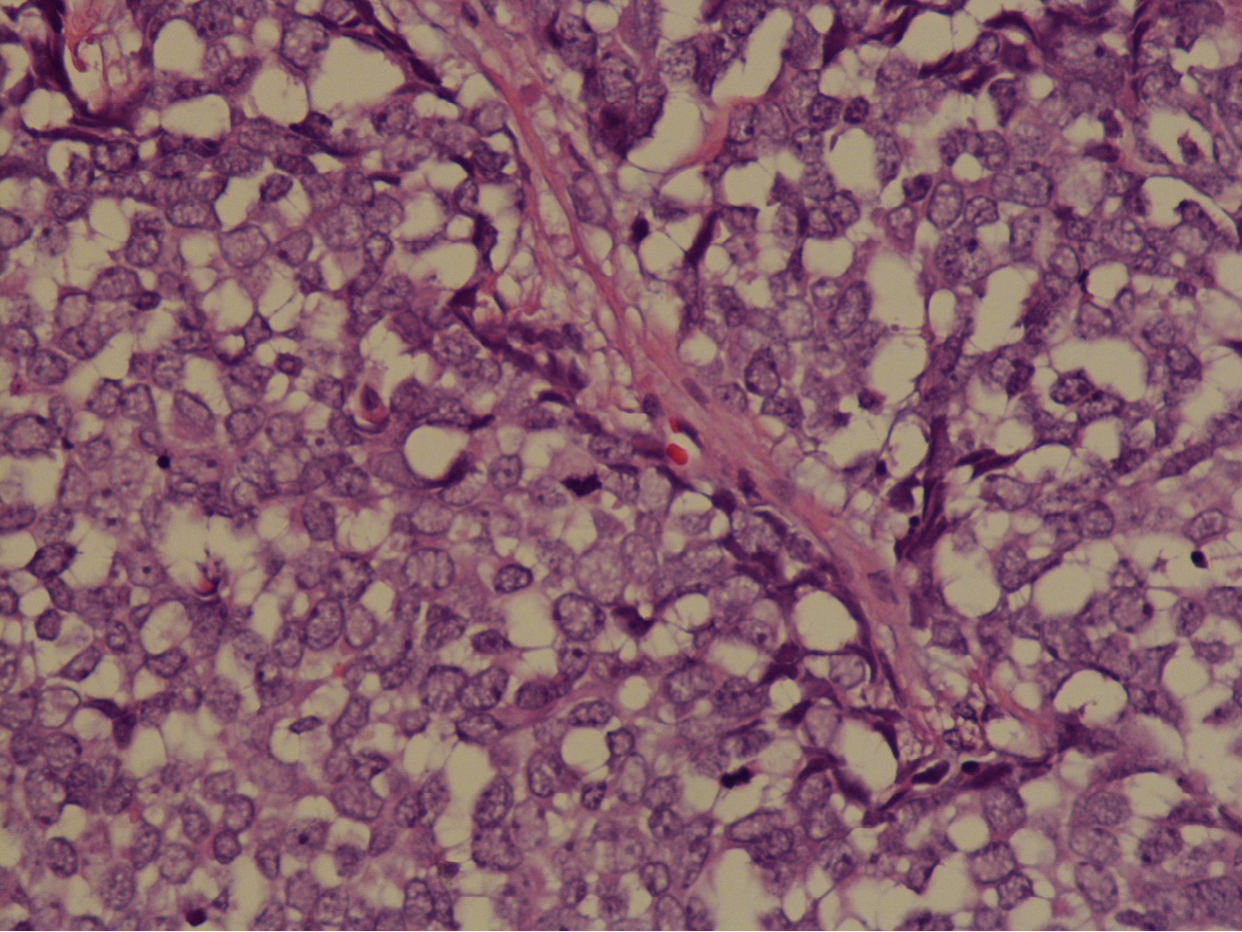
Histopathology (high resolution) showing neuroendocrine features of the tumor.

**Figure 3:**
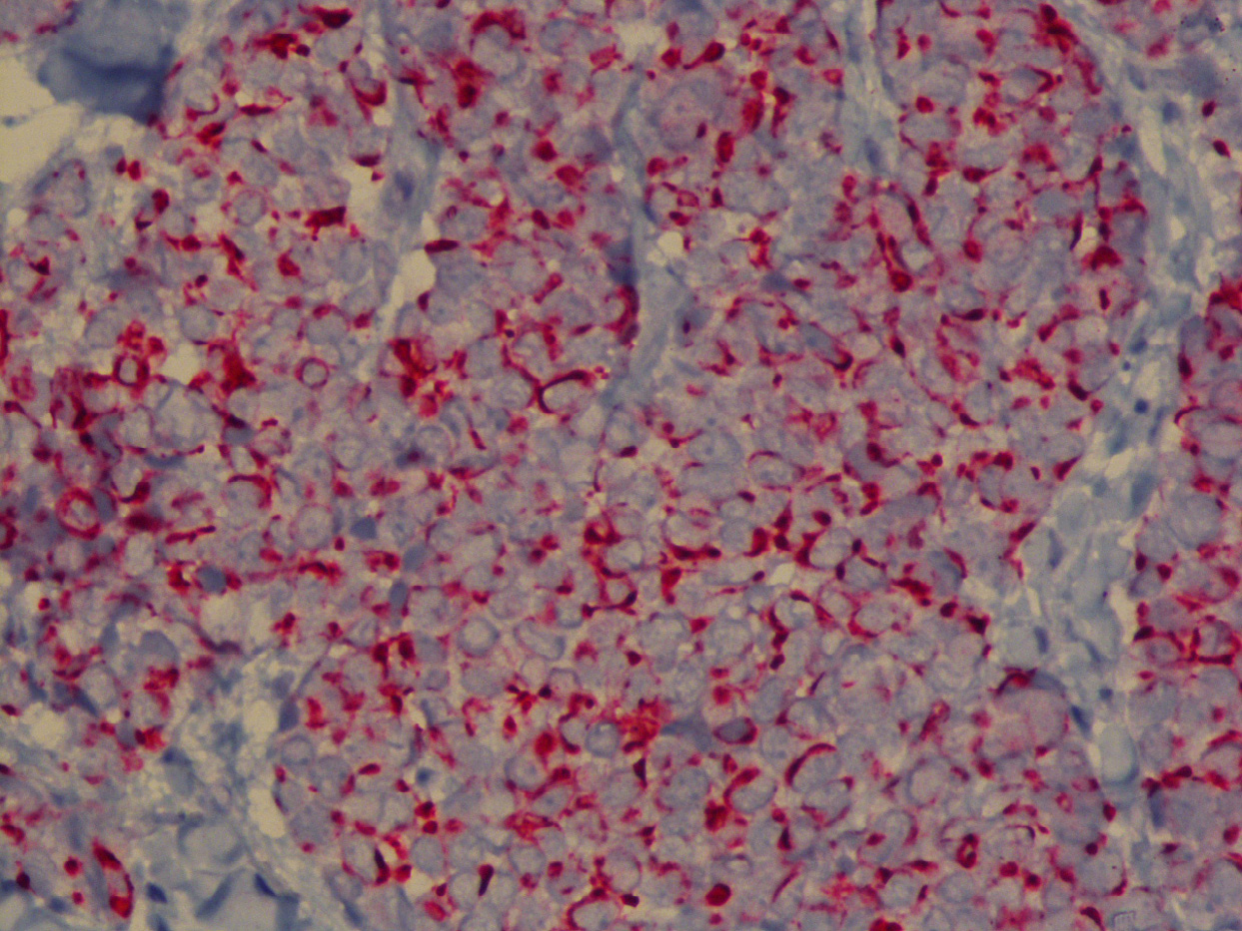
Immunohistochemistry showing positive cytokeratin 20 (CK 20) stain.

**Figure 4:**
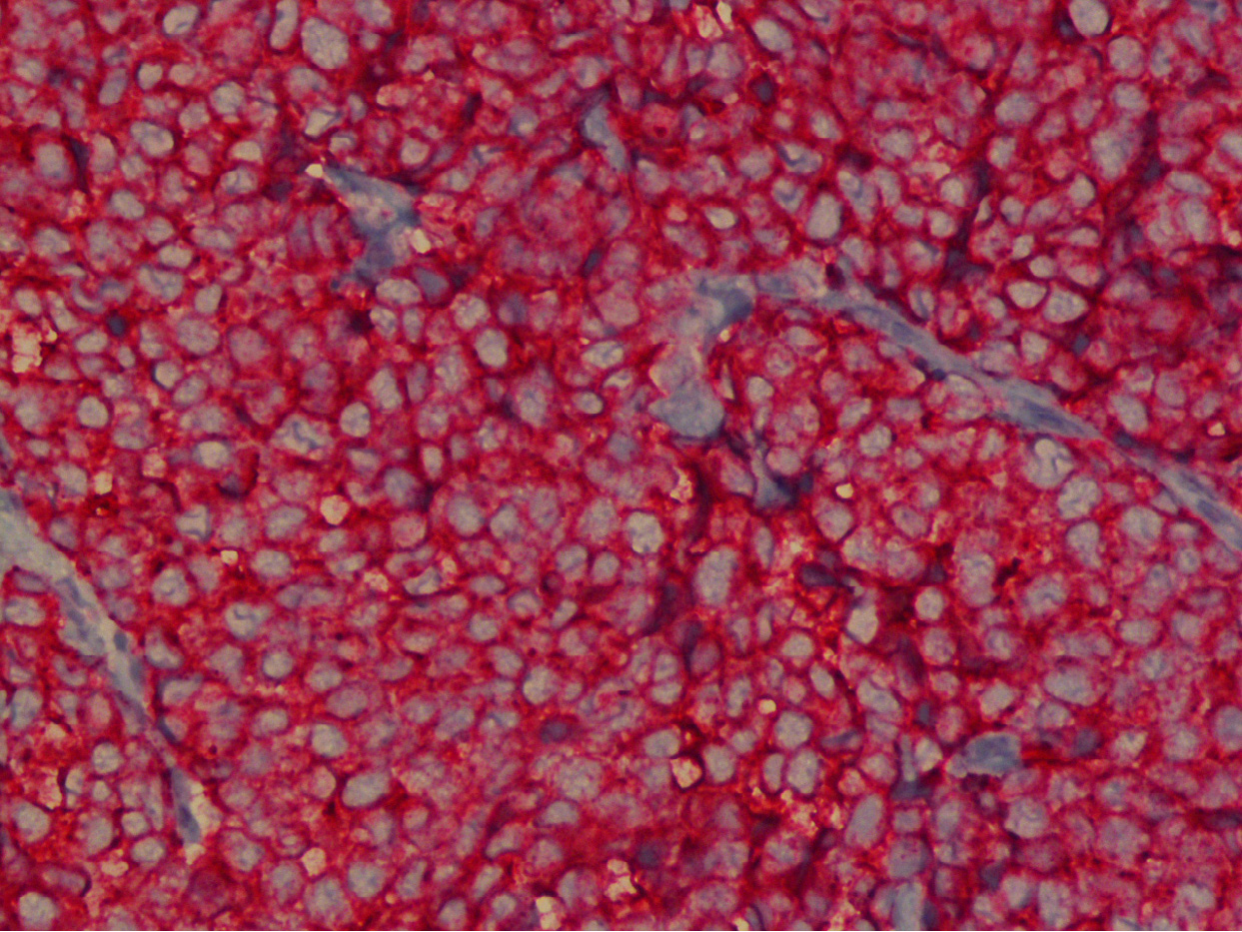
Immunohistochemistry showing positive synaptophysin stain.

This is an 83‐year‐old lady who presented to her primary care physician with a small lump on her right cheek. Review of systems was otherwise negative. There was no evidence of pathological lymph nodes in mandibular region or cervical lymph nodes. She was referred to a dermatologist who performed excision. Histopathology showed primary neuroendocrine carcinoma (Figs 1 and 2). The depth of invasion was 5.5 mm; there was more than 10 mitoses/sq mm. Initially, the margins were positive, so wider excision was performed to achieve negative margins. Sentinel lymph node biopsy was negative. Immunohistochemical stains showed tumor to be positive for cytokeratin 20, cytokeratin AE1/AE3, and synaptophysin (Figs 3 and 4). The above findings were consistent with Merkel cell carcinoma. Positron emission tomography‐computed tomography (PET/CT) was unremarkable. The Merkel polyomavirus serology test (AMERK test) came back positive for polyomavirus antibodies. Adjuvant radiation therapy was discussed with the patient; however, she declined it and chose observation. Merkel cell carcinoma is a rare neoplasm that accounts for less than 1% of cutaneous malignancies. The various differentials are basal cell carcinoma, lymphoma, and metastatic small cell cancer of lung. It is an aggressive primary cutaneous neuroendocrine carcinoma with cytoplasmic, dense‐core neuroendocrine granules, and keratin filaments. It usually presents as red, purple, or violaceous firm painless nodule or plaque frequently in head and neck regions, with the periorbital region being the most common site of occurrence. Merkel cell carcinoma (MCC) causes local and distant metastases with high rates of recurrence. Paranuclear dot‐like positivity for cytokeratin (CK) 20 is a fairly specific and sensitive marker for MCC; most of the case are CK 20‐positive and CK 7‐negative 1, 2. MCC is also known to express synaptophysin and AE1/AE3 2. Treatment depends on the clinical stage at presentation. Wide local excision with a clearance margin of 3‐5 cm is recommended for clinically node‐negative patients. Adjuvant radiotherapy along with nodal dissection is recommended for patients with clinical nodal disease 3, 4. More than 20% of all cancers worldwide are virus‐associated and are characterized by robust immune infiltrates and PD‐L1 expression. Thus, pembrolizumab and nivolumab targeting PD‐1 receptor can be used for its treatment 5.

## Authorship

MPR: wrote the case description. PB and JS: contributed to the description on Merkel cell carcinoma and the key clinical message. EBM: provided the description of the pathology images.

## Conflict of Interest

None declared.
